# Plasma‐activated media selectively induces apoptotic death via an orchestrated oxidative stress pathway in high‐grade serous ovarian cancer cells

**DOI:** 10.1002/1878-0261.13768

**Published:** 2024-12-03

**Authors:** Lorena T. Davies, Raja Ganesen, John Toubia, Sung‐Ha Hong, Sushil Kumar KC, Martin K. Oehler, Carmela Ricciardelli, Endre J. Szili, Nirmal Robinson, Melissa R. Pitman

**Affiliations:** ^1^ Centre for Cancer Biology University of South Australia and SA Pathology Adelaide Australia; ^2^ Future Industries Institute University of South Australia Adelaide Australia; ^3^ Reproductive Cancer Research Group; Discipline of Obstetrics and Gynaecology, Adelaide Medical School The University of Adelaide Australia; ^4^ Robinson Research Institute Adelaide Australia; ^5^ Department of Gynaecological Oncology Royal Adelaide Hospital Australia; ^6^ Adelaide Medical School The University of Adelaide Australia; ^7^ School of Biological Sciences The University of Adelaide Australia

**Keywords:** apoptosis, cold atmospheric pressure, ovarian cancer, oxidative stress, plasma‐activated media

## Abstract

High‐grade serous ovarian cancer (HGSOC) is the most common and aggressive type of ovarian cancer. Due to a lack of an early detection test and overt symptoms, many patients are diagnosed at a late stage where metastasis makes treatment very challenging. Furthermore, the current standard treatment for HGSOC patients, consisting of debulking surgery and platinum‐taxane chemotherapy, reduces quality of life due to debilitating side‐effects. Sadly, 80–90% of patients diagnosed with advanced stage ovarian cancer will die due to treatment resistance. As such, novel therapeutic strategies for HGSOC that are both more effective and less toxic are urgently required. Here we describe the assessment of cold atmospheric pressure (CAP) gas discharge technology as a novel treatment strategy in pre‐clinical models of HGSOC. Plasma‐activated media (PAM) was generated using cell growth media. HGSOC cell lines, patient ascites cells and primary tissue explants were tested for their response to PAM via analysis of cell viability, cell death and oxidative stress assays. Our data show that PAM treatment can be more effective than standard carboplatin chemotherapy at selectively targeting ovarian cancer cells in primary patient samples. Further, we also observed PAM to induce apoptosis in HGSOC cancer cell lines via induction of oxidative stress and mitochondrial‐mediated apoptosis. These findings suggest that PAM is a viable therapeutic strategy to test in *in vivo* models of ovarian cancer, with a view to develop an intraperitoneal PAM‐based therapy for HGSOC patients. Our studies validate the ability of PAM to selectively target tumour tissue and ascites cells. This work supports the development of PAM towards *in vivo* validation and translation into clinical practice.

## Graphical abstract

High‐grade serous ovarian cancer (HGSOC) is the most common and aggressive type of ovarian cancer with urgent need for new treatment strategies. This study evaluates plasma‐activated media (PAM) as a novel treatment for HGSOC, showing its ability to selectively induce oxidative stress and apoptosis in HGSOC cell lines, patient ascites cells and primary tissue explants.

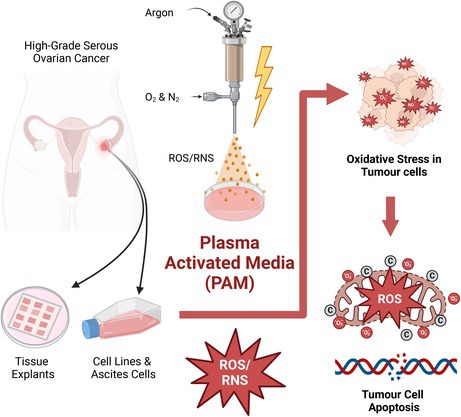

**Abbreviations**
ATCC: American Tissue Culture Collection
BRCA: breast cancer gene
CAP: cold atmospheric pressure
CBP: carboplatin
EOC: epithelial ovarian cancer
HGSOC: high‐grade serous ovarian cancer
HRP: horseradish peroxidase
KRAS: Kirsten Rat sarcoma viral oncogene homologue
MOMP: mitochondrial outer membrane permeabilization
NRF2: nuclear factor erythroid‐derived 2‐like 2
OPD: ortho‐phenylenediamine
PAM: plasma‐activated media
RONS: reactive oxygen and nitrogen species
ROS: reactive oxygen species
RRID: research resource identifiers
TMRE: tetramethylrhodamine
TP53: tumour protein 53

## 1 Introduction

Ovarian cancer is the seventh most common cancer globally in women and has a 5‐year survival rate of only about 50% [1, 2]. High‐grade serous ovarian cancer (HGSOC) is the most prevalent subtype and comprises approximately 75% of all ovarian cancer cases [3]. Nearly all HGSOCs harbour mutations in TP53. Germline BRCA1/2 mutations are identified in 13–15% of ovarian cancers, while an additional 5–7% of ovarian cancers have somatic BRCA1/2 mutations [4]. With vague early symptoms and no early detection testing, HGSOC is often diagnosed at late stage where disease has metastasised. Despite best efforts to remove tumour load by debulking surgery and adjuvant chemotherapy, most patients develop chemotherapeutic resistance and subsequently succumb to the disease.

Cold (non‐thermal) atmospheric pressure ionised gas (plasma) (CAP) is emerging as a new technology to treat cancers [5]. CAP works by generating a high‐energy glow discharge at biocompatible (around body) temperature, which interacts with the ambient air to produce a rich mixture of reactive oxygen species (ROS) and reactive nitrogen species (RNS), collectively known as RONS [6]. When the CAP is directed at the body the RONS are delivered into the biological fluid where they can exert oxidative stress on cells [7]. Cancer cells are more vulnerable to oxidative stress because malignancy is often accompanied by a reduced level of membrane cholesterol making the cells more susceptible to lipid peroxidation and subsequently RONS ingress [8]. Furthermore, higher basal levels of oxidative stress make the cells more vulnerable to ROS insults [9, 10], and highly proliferative cancer cell populations synthesise more DNA making it more likely to be damaged through oxidation [5]. A major advantage of CAP compared to therapies such as ionising radiation, is that CAP is non‐ionising and therefore is much less damaging to surrounding healthy cells and tissue. There is growing evidence suggesting that CAP‐based treatments selectively target destruction of cancers without affecting normal (healthy) cells and tissue [11, 12, 13, 14]. However, a major limitation of CAP is that it requires delivering the CAP energy via a direct path and in close proximity to the tissue, which makes it difficult to treat invasive and dispersed tumours. This problem is also associated with HGSOC that follows a well‐characterised pattern of spread; the cancer cells tend to disperse throughout the peritoneal cavity attaching to organ surfaces [15].

The problem of reaching all ovarian cancer cells throughout the peritoneal cavity can be overcome by using a plasma‐activated media (PAM). PAM is generated by exposing liquid media to CAP that produces a liquid formulation of RONS, including hydrogen peroxide (H_2_O_2_), hydroperoxyls (HO_2_), hydroxyls (OH), nitric oxides (NO_x_), and other oxidising agents such as molecular oxygen (O_2_) [16]. The heterogeneous mix of RONS in PAM is thought to produce the favourable anticancer effects not easily attainable by other chemotherapy methods [13]. PAM can potentially be used to wash the peritoneal cavity post‐surgery or be injected in accordance with other established techniques such as hyperthermic intraperitoneal chemotherapy (HIPEC) [17]. Although intraperitoneal chemotherapy has proven to be effective, it is poorly tolerated due to neurotoxicity and nephrotoxicity side‐effects [18]. This has limited the widespread adoption of intraperitoneal chemotherapy. In this regard, PAM could make a difference by offering a potentially equally (if not more) effective yet more tolerable form of intraperitoneal chemotherapy.

Previous studies have demonstrated the high efficacy of PAM against ovarian cancer cells [19, 20, 21]. However, PAM therapy has yet to be assessed in genetically representative HGSOC models. Here, PAM is investigated as a novel treatment approach for HGSOC, utilising representative cell lines and primary HGSOC patient tumour tissues and ascites cells. The study specifically focuses on elucidating the oxidative stress pathways involved in the PAM‐mediated pro‐apoptotic cell death in tumour cells and tissues. The results of this research might find use in the future development of PAM as standalone or adjunct therapy for HGSOC.

## 2 Materials and methods

### 2.1 Generation and characterisation of PAM

**Fig. 1:**
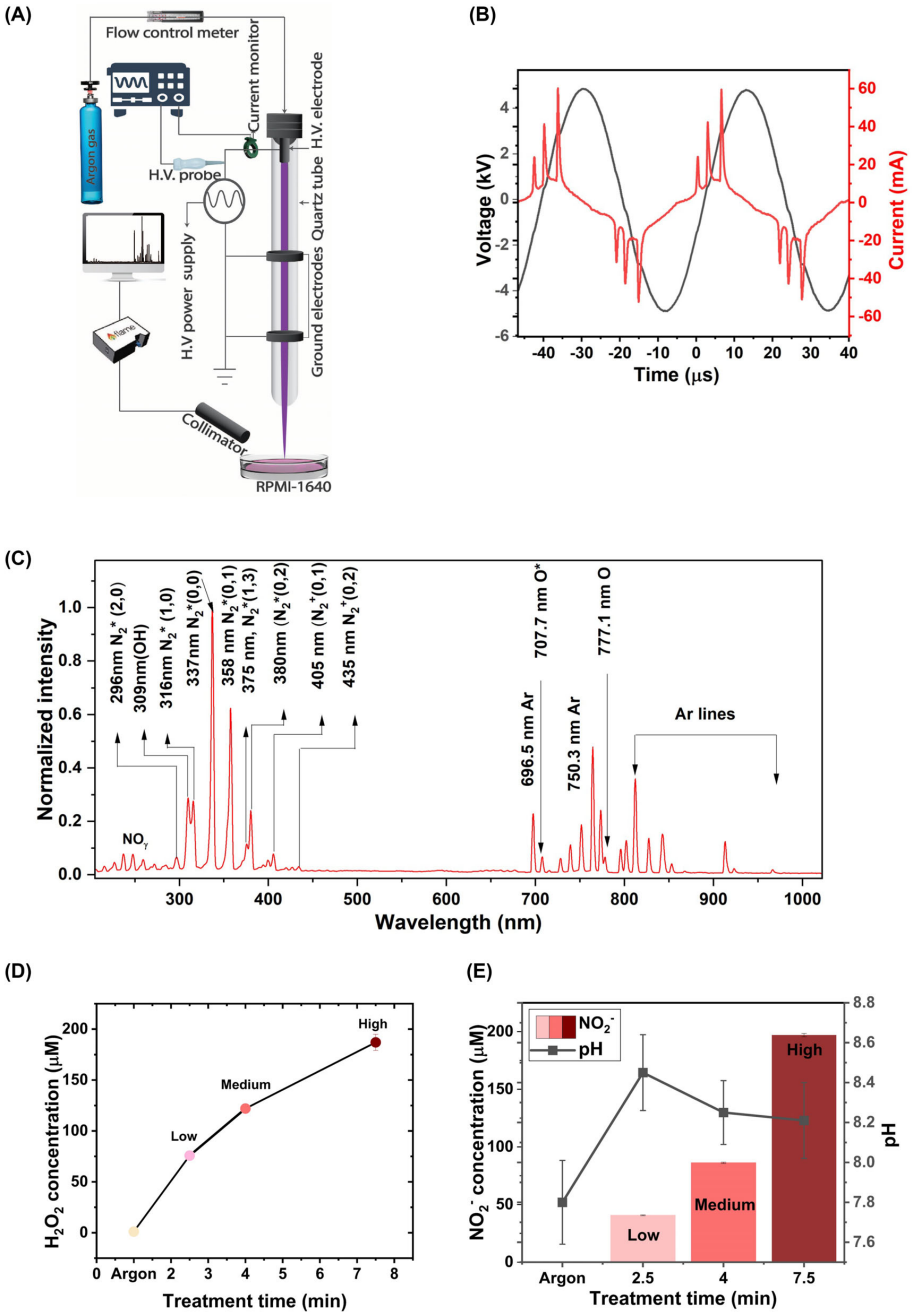
Production and characterisation of Plasma‐Activated Media (PAM). (A) Experimental setup of the plasma jet used to prepare PAM from RPMI‐1640 media (not drawn to scale). The approximate location of the electrical (High voltage (HV) probe and current monitor) and optical (emission spectroscopy) sampling points are shown in the figure. (B) An example voltage and current waveform trace during production of PAM acquired from an average of 128 waveforms with 5 giga samples per second (GSPS) sampling rate. (C) An example optical emission profile during production of PAM acquired with the spectrometer set at an integration time of 100 ms (*n* = 1). (D) H_2_O_2_ concentration in PAM (*n* = 3). (E) ${\mathrm{NO}}_{2}^{-}$ concentration and pH in low‐, medium‐ and high‐dose PAM (*n* = 3). In (D) and (E) RPMI‐1640 treated with argon (Ar) flow only (i.e., with no applied voltage and the CAP jet off) is used as the negative control. Data is represented as mean ± standard error of the mean (SEM). Graphs were generated using Origin origin pro (Origin Lab, Northhampton, MA, USA).

The CAP device and experimental setup used to prepare PAM is shown in Fig. 1A. The setup and protocols to characterise its electrical and optical characteristics have been previously described [22]. The CAP device consists of a high voltage (HV) electrode (hollow, stainless‐steel tube with inner diameter = 0.4 mm, outer diameter = 0.8 mm and length = 15 mm) inserted inside a quartz tube (inner diameter = 2 mm, outer diameter = 4 mm and length = 100 mm). Two annular copper electrodes were positioned at 35 and 165 mm below the tip of the high voltage electrode. Two ground electrodes were used to operate the plasma jet because this was previously shown to enhance the production of ROS whilst maintaining a low gas temperature [23]. High purity (99.9999%) argon at a pressure of 40 psi was purged through the HV electrode with a flowmeter (Omega Engineering, Norwalk, CT, USA, Model # FLDAR3501C) at 0.8 standard litres per minute (SLPM). A voltage of 11 kVp‐p at 23.5 kHz was applied to the HV electrode with an AC power supply (PVM500, Information Unlimited, Mont Vernon, NH, USA). Under these operating conditions a CAP jet was launched into the ambient air and directed onto the target RPMI‐1640 (Gibco/Thermo Fisher Scientific, Waltham, MA, USA) cell culture medium. All treatments were performed at 5 mm between the orifice of the quartz tube and the surface of the cell culture medium. CAP treatments were performed for 2.5, 4 and 7.5 min with the CAP remaining in contact with media throughout all treatments. Cell culture medium treated with the argon gas only (i.e., with no applied voltage) for 7.5 min was used as a control. Following treatment, RPMI‐1640 was supplemented with 10% heat‐inactivated foetal bovine serum (GE Healthcare Life Sciences, Chicago, IL, USA) and 1% penicillin–streptomycin (Gibco) before administration to cells and explants.

A HV probe (Pintek Electronics, New Taipei City, Taiwan, Model # HVP39pro) and current monitor (Pearson Electronics, Palo Alto, CA, USA, Model # 8585c) were used to measure the electrical signals during treatment of the cell culture medium as shown in Fig. 1A. The optical emission from the CAP jet was measured using a spectrometer (Ocean Optics, Orlando, FL, USA, Flame T‐XR1‐ES) equipped with an optical fibre of diameter 600 μm and collimating lens. The integration time was set to 100 ms with averaging kept at 1. The distance between the CAP jet and the optical fibre was maintained at 10 mm. All spectra were acquired using the cosine corrector (CC‐3‐UV‐S) attached to the front end of solar‐resistant optical fibre with diameter 600 μm to collect light evenly from all angles. The background spectrum was acquired in the dark.

### 2.2 H_2_O_2_ detection

The concentration of H_2_O_2_ in PAM was measured by following its reaction with ortho‐phenylenediamine (OPD) in the presence of horseradish peroxidase (HRP) as previously described [22]. OPD is a colourless compound that reacts with H_2_O_2_ in the presence of HRP to form a yellow‐coloured compound called 2–3‐diaminophenazine that has an absorbance maximum of 450 nm. H_2_O_2_ concentration was quantified from a calibration curve using known concentrations of H_2_O_2_. Experiments were performed by first preparing a mixture of 18.5 mm ortho‐OPD powder (Sigma‐Aldrich, St Louis, MO, USA, catalogue # P5412) and 4 μg·mL^−1^ HRP (Sigma‐Aldrich, catalogue # P6782) dissolved in 10 mL of water. Then, 100 μL of the OPD‐HRP solution was added to wells of a 96‐well plate containing 100 μL of PAM or a known concentration of H_2_O_2_. The plate was incubated at ambient temperature for 30 min in the dark. The absorbance of the solutions was measured at a wavelength of 450 nm using a plate reader (SpectraMax M2, Molecular Devices, San Jose, CA, USA).

### 2.3 NO_2_^−^ detection

${\mathrm{NO}}_{2}^{-}$ concentration was calculated by measuring the oxidation of Griess reagent as previously described [22]. In the presence of ${\mathrm{NO}}_{2}^{-}$, Griess reagent is oxidised into a diazonium salt, which readily couples with *N*‐(1‐naphthalenediamine) to form a highly coloured azo dye with an absorbance maximum of 492 nm. ${\mathrm{NO}}_{2}^{-}$ concentration was quantified from a calibration curve using known concentrations of ${\mathrm{NO}}_{2}^{-}$. A stock of Griess reagent (Sigma‐Aldrich, Catalogue # 6000‐43‐7) was prepared by dissolving 50 mg of Griess reagent per 1 mL of water. A 100 μL aliquot of the PAM was mixed with 100 μL of the Griess reagent stock in a 96‐well plate and incubated for 30 min in the dark before measuring the absorbance of the solution on the microplate reader.

### 2.4 HGSOC and peritoneal cell culture

Cell lines were purchased from the American Tissue Culture Collection (ATCC); OV90 (ATCC:CRL‐11732, RRID:CVCL_3768) and CAOV3 (ATCC:HTB‐75, RRID:CVCL_0201). Both cell lines were authenticated (2024) by short tandem repeat testing (100% match) using the GenePrint‐10 System (Australian Genomics Research Facility (AGRF), Brisbane, Australia). Cells were maintained in RPMI‐1640 (OV90, Life Technologies/Thermo Fisher Scientific, Waltham, MA, USA) or DMEM (CAOV3, Life Technologies/Thermo Fisher Scientific), 10% FBS (GE Healthcare Life Sciences/Thermo Fisher Scientific), 1% penicillin/streptavidin (P/S, Gibco) at 37 °C 5% CO_2_ for a maximum of 25 passages and were performed with mycoplasma‐free cells. LP‐9 peritoneal cells were purchased from Coriell Cell Repositories (Camden, NJ, USA). All cells were treated with argon or PAM‐treated RPMI‐1640 media.

### 2.5 Cell viability assay (MTS)

Cells were seeded in growth media (5000 cells per well) in 96‐well plates 24‐h prior to treatment. Growth media was replaced with PAM, argon‐treated control (argon‐treated), or fresh growth media. For rescue experiments vehicle (DMSO) or reagents were added 1 h prior to PAM treatment and included with PAM treatment or as indicated in the figure legend. Rescue reagents: Pan‐caspase inhibitor (Q‐VD‐OPh hydrate (QVD), Merck SML0063), Oxidative stress (Cell permeable Glutathione Ethyl Ester (GEE), Cayman Chemical, Ann Arbor, MI, USA, #14959), Lipid peroxidation/Ferroptosis Inhibitor (Ferrostatin‐1, Merck, SML0583). Cell viability was quantified using the CellTiter 96® AQueous Non‐Radioactive Cell Proliferation Assay (MTS reagent: 3‐(4,5‐dimethylthiazol‐2‐yl)‐5‐(3‐carboxymethoxyphenyl)‐2‐(4‐sulfophenyl)‐2H‐tetrazolium) according to the manufacturer's instructions (Promega, Madison, WI, USA). Cell images were taken at 10× magnification using an EVOS XL (Life Technologies) at the indicated times.

### 2.6 Flow cytometry

Cells were seeded into 6‐well plates (0.25 × 10^6^ per well) 24 h prior to treatment. Growth media was replaced with PAM or the control (argon‐treated). Cells were harvested using trypsin and combined with cells pelleted from the growth media, stained according to the manufacturer's instructions and analysed by flow cytometry (BD Biosciences, Franklin Lakes, NJ, USA, LSRFortessa). For quantification of mitochondrial membrane potential (∆ᴪm), cells were stained with Tetramethylrhodamine (TMRE, Invitrogen/Thermo Fisher Scientific, Waltham, MA, USA, # T669, excitation 549/emission 574 nm). For quantification of apoptosis, cells were stained with Annexin V‐FITC (BD Biosciences, Franklin Lakes, NJ, USA, #556419, Annexin binding buffer ##556454, excitation 498/emission 517 nm) and Propidium Iodide (PI) (0.1 μm final, Sigma‐Aldrich #P1450, excitation 535/emission 617 nm) or Nucview (Biotium, Fremont, CA, USA, #10402, 5 μm final, excitation 500/emission 530 nm).

### 2.7 Western blot analysis

Cells were seeded in 60 mm dishes (1 × 10^6^ cells per well) 24 h prior to treatment. Growth media was replaced with PAM treatment and incubated for the indicated times. Cells were washed with phosphate‐buffered saline (PBS) and then scraped directly into Lysis buffer: 10 mm Tris/HCl pH 7.4, 137 mm NaCl, 10% glycerol, 1% NP40, 10 mm beta‐glycerophosphate, 2 mm sodium vanadate (activated), 2 mm sodium fluoride, 10 mm sodium pyrophosphate, 1× protease inhibitor cocktail (Roche, Basel, Switzerland, #5056489001). Equal quantity of protein was analysed by SDS/PAGE, transferred to nitrocellulose and analysed by immunoblotting with the following antibodies. PARP‐1 (Cell Signaling Technology (CST), Danvers, MA, USA, #9542), Phospho‐NRF2 (Ser40) (Invitrogen/Thermo Fisher Scientific, #PA5‐67520), Phospho‐histone H2A.X (Ser139) (CST, Danvers, MA, USA, #9718), Alpha‐Tubulin (Abcam, Cambridge, UK, #ab7291).

### 2.8 Tumour explant preparation

Tissue explants were prepared as per previous studies [24]. Briefly, collection of ovarian cancer tissues and the study methodologies were approved by the Royal Adelaide Hospital Human Ethics Committee. Samples were collected at the Royal Adelaide Hospital (South Australia) between March 2018 and May 2021 (ethics approval HREC/17/RAH/13 and HREC/18/CALHN/811; Patient information provided in Table S1). The experiments were undertaken with the understanding and written consent of each subject and the study methodologies conformed to the standards set by the Declaration of Helsinki. Cryopreserved tissue fragments were thawed into media (RPMI 10% FBS and 1% P/S), cut into 1 mm^3^ pieces, and 3–4 pieces were grown on pre‐soaked gelatin dental sponges (Spongostan, Johnson & Johnson, New Brunswick, NJ, USA) in 24‐well plates. A sample of each patient tissue was preserved immediately in RNA‐later for molecular characterisation by RNA‐sequencing analysis. Thawing media was removed from the explants and the following treatments were added (0.5 mL, in duplicate) argon control, PAM (high dose), carboplatin (CBP) (100 μm) or vehicle (DMSO, 0.1% final). Tissues were grown in a humidified atmosphere at 37 °C containing 5% CO_2_ and fixed for immunohistochemical analysis 72 h post‐treatment.

### 2.9 Immunohistochemistry

Tissue sections were processed for immunohistochemistry as described previously [24]. Tissue sections on slides were incubated overnight at 4 °C with rabbit monoclonal antibody to rabbit polyclonal antibody to cleaved caspase‐3 (1 : 200, Cat # 9661, Cell Signaling Technology, Danvers, MA, USA) or rabbit monoclonal phospho‐histone H2A.X (1 : 500, Cat # 9718, Cell Signaling Technology). Visualisation of immunoreactivity was achieved using biotinylated anti‐rabbit or anti‐mouse immunoglobulins, streptavidin‐peroxidase conjugate and diaminobenzidine substrate. Slides were counterstained with haematoxylin and eosin (H&E) (Sigma‐Aldrich), dehydrated and mounted in Pertex (Medite Medizintechnik, Germany) and digitally scanned using the NanoZoomer (Hamamatsu Phontonics K.K, Hamamatsu, Japan). Cancer tissue areas (5–8 images per treatment group) were randomly selected across the tissue fragments in the cancer tissues and stromal fibroblasts and the number of positive cleaved caspase‐3 cells per mm^2^and phospho‐histone H2A.X cells per mm^2^ were quantitated in tumour regions and stromal regions using qupath software (Version 0.4.3) [25] in a blinded fashion identified in serial H&E stained sections. Data is expressed as % of vehicle or argon control.

### 2.10 Tumour ascites samples

Samples were collected at the Royal Adelaide Hospital (South Australia) between March 2018 and May 2021 (ethics approval HREC/18/CALHN/811; Patient information provided in Table S1). Samples were prepared as previously described [26]. Briefly, 50 mL of primary patient ascites sample was centrifuged at 500 × g for 10 min (room temperature). The cell pellet was resuspended in growth media SensiCellTM RPMI 1640 (Gibco) supplemented with 4 mm l‐glutamine, 10% FBS (Sigma‐Aldrich) and antibiotics (100 U penicillin G, 100 μg·mL^−1^ streptomycin sulfate and 100 μg·mL^−1^ amphotericin B (Sigma‐Aldrich)). Primary ovarian cancer cell lines were maintained in growth media and cryopreserved and stored in liquid nitrogen at passage 2. Primary cells were used between passages 3 and 5 for the described experiments. Patient clinico‐pathological characteristics are shown in Table S1. Genomic DNA and RNA was extracted from the ascites cells for molecular characterisation.

### 2.11 Molecular characterisation of tissue explants and ascites samples

Tissue explant RNA was extracted according to manufacturer's instructions (Qiagen, Hilden, Germany). PolyA+ enriched RNA‐seq libraries from explant and tumour ascites were multiplexed and sequenced on the Illumina NextSeq 500 platform using the stranded, single end protocol with a read length of 70 nt. Raw data, averaging 35 million reads per sample were analysed and quality checked using the FastQC program (http://www.bioinformatics.babraham.ac.uk/projects/fastqc). Reads were mapped against the human reference genome (hg38) using the STAR spliced alignment algorithm [27] (version 2.7.7a with default parameters and ‐‐chimSegmentMin 20, ‐‐quantMode GeneCounts) returning an average unique alignment rate of 88%. Alignments were visualised and interrogated using the Integrative Genomics Viewer v2.3.80 [28] (Table S2). Classification of samples into molecular subtypes was carried out using the gene set variation analysis (GSVA) r package [29] (version 1.51.6) against the gene collections derived from the Predictor of High‐grade Serous Ovarian Carcinoma Molecular SubTYPE (PrOTYPE) study [30] (Table S3). Mutation detection was conducted using freebayes variant detection algorithm [31] (version 1.3.7) against a subset of 523 human cancer‐associated genes. Inference on the potential effect of the identified mutations was achieved using the Ensembl Variant Effect Predictor [32] (Tables S4 and S5).

### 2.12 Statistical analysis

Data are presented as mean ± standard deviation (SD) or ± standard error of the mean (SEM) and were analysed using graphpad prism (version 9, GraphPad, La Jolla, CA, USA) unless otherwise specified. Statistical significance was evaluated by one‐way analysis of variance (ANOVA), followed by Tukey's multiple comparisons test. *P* < 0.05 was considered statistically significant.

## 3 Results

### 3.1 Overview of CAP treatment and oxidative properties of PAM

A CAP jet was used to treat RPMI‐1640 medium to prepare PAM (Fig. 1A). The electrical property of the CAP is characterised by three prominent current peaks seen in the current waveforms (Fig. 1B). The first two current peaks are associated with the plasma discharge between the high voltage electrode to the first and second ground electrodes (Fig. 1A). The third current peak is produced when the CAP jet touches the RPMI‐1640, indicating strong electrical contact during treatment. This strong electrical contact facilitates efficient production of RONS through electron dissociation reactions, as previously described [23]. In addition, several high‐energy state species are generated during the CAP jet treatment of the RPMI‐1640 (Fig. 1C). These include high‐energy state oxygen, nitrogen and argon species seen in the optical emission spectra of the CAP jet from the ultra‐violet to the visible wavelength range (Fig. 1C). These high‐energy state species can form RONS through a variety of chemical processes [6]. The concentrations of H_2_O_2_ and ${\mathrm{NO}}_{2}^{-}$ in PAM were measured to estimate the total ROS and RNS, respectively, as previously described [33] (Fig. 1D,E). The H_2_O_2_ concentrations in the PAM were 76, 122 and 187 μm, which were prepared with 2.5, 4 and 7.5 min of CAP jet treatment. These formulations were arbitrarily categorised in corresponding order as low, medium and high dose, since H_2_O_2_ is a major oxidant with a long biological half‐life in PAM. ${\mathrm{NO}}_{2}^{-}$ concentration was measured at 41, 86 and 197 μm in low, medium and high‐dose PAM, respectively. Whilst ${\mathrm{NO}}_{2}^{-}$ has a relatively low level of reactivity compared to H_2_O_2_, it is readily generated as an end‐product in PAM from reactions of more reactive RNS that cannot be as easily or reliably measured such as nitric oxide (NO) and peroxynitrite (ONOO^−^). In addition, ${\mathrm{NO}}_{2}^{-}$ is also thought to enhance the anticancer effects of other agents in PAM such as H_2_O_2_ [34]. The pH in all three PAM preparations remained constant at around pH 8.

### 3.2 PAM reduces viability in HGSOC cell lines

**Fig. 2:**
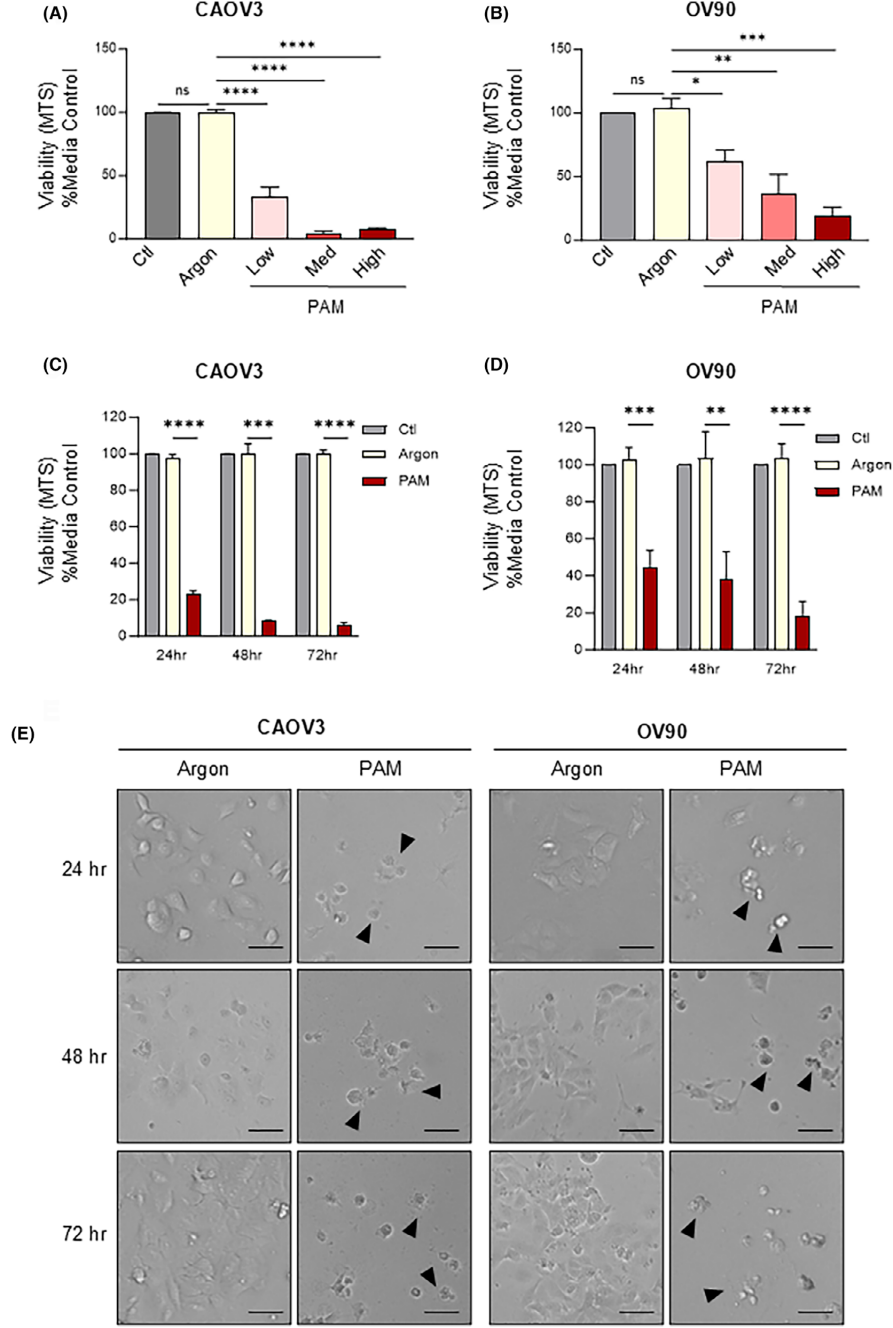
Plasma‐Activated Media (PAM) reduces cell viability in High‐Grade Serous Ovarian Cancer Cell (HGSOC) cell lines. HGSOC cells (A) CAOV3 or (B) OV90 cells were treated with control (Ctl), argon‐treated (Argon) or low‐, medium‐ or high‐dose PAM and the cell number was quantified using 3‐(4,5‐dimethylthiazol‐2‐yl)‐5‐(3‐carboxymethoxyphenyl)‐2‐(4‐sulfophenyl)‐2H‐tetrazolium (MTS) cell viability assay after 72 h. (C) CAOV3 or (D) OV90 cell were treated with a high‐dose PAM and cell numbers were quantified by MTS after 24, 48 and 72 h. (E) Representative images of cells treated with either argon or high‐dose PAM for 24, 48 and 72 h at 10× (*n* = 2). Black arrows indicate cells displaying characteristics of programmed cell death. Scale bar indicates 100 μm. MTS results are normalised to % media control and represented as mean ± Standard Deviation for three independent experiments (*n* = 3). Significance was determined by one‐way ANOVA with multiple comparisons (graphpad, prism v9). * = *P* < 0.05, ** = *P* < 0.01, *** = *P* < 0.001, **** = *P* < 0.0001, ns, not significant.

To test the effect of PAM on the viability HGSOC, the three doses of PAM (low, medium and high) were tested on two HGSOC‐representative cells CAOV3 and OV90. Cells were treated with control media, argon‐treated or PAM at varying doses and the number of viable cells was quantified by MTS assay at 72 h (Fig. 2A,B). While the argon treatment had no significant effect compared to the media control, all doses of the PAM treatment significantly decreased the cell viability in both the CAOV3 and OV90 cells (Fig. 2A,B). The OV90 cells were dose‐dependently impacted by the increasing doses of PAM (38%, 63% and 83% decrease in cell viability) (Fig. 2B). In contrast, CAOV3 cells were affected to a similar extent by the two higher doses of PAM (93–95% decrease in viability) (Fig. 2A), indicating greater sensitivity to the treatment than the OV90 cells.

To explore the time‐dependent effects of the PAM treatment, cell viability was measured up to 72 h following treatment with high‐dose PAM. After 24 h, viability of CAOV3 and OV90 cells was decreased by 75% and 50%, respectively (Fig. 2C,D). Cell viability decreased further at 48‐ and 72‐h timepoints. To track any morphological changes induced by PAM treatment, images of the cells were taken over the three timepoints (Fig. 2E). While the cell population increased up to 72 h in the argon‐treated control group, a noticeable decline in cell number was observed for the PAM‐treated group (Fig. 2E). In line with the decreased cell viability and lack of recovery over time, the morphology of the cells was drastically altered and cells appeared to display membrane blebbing and cell shrinkage that would indicate a programmed cell death, such as apoptosis.

### 3.3 PAM induces apoptosis in HGSOC cell lines

**Fig. 3:**
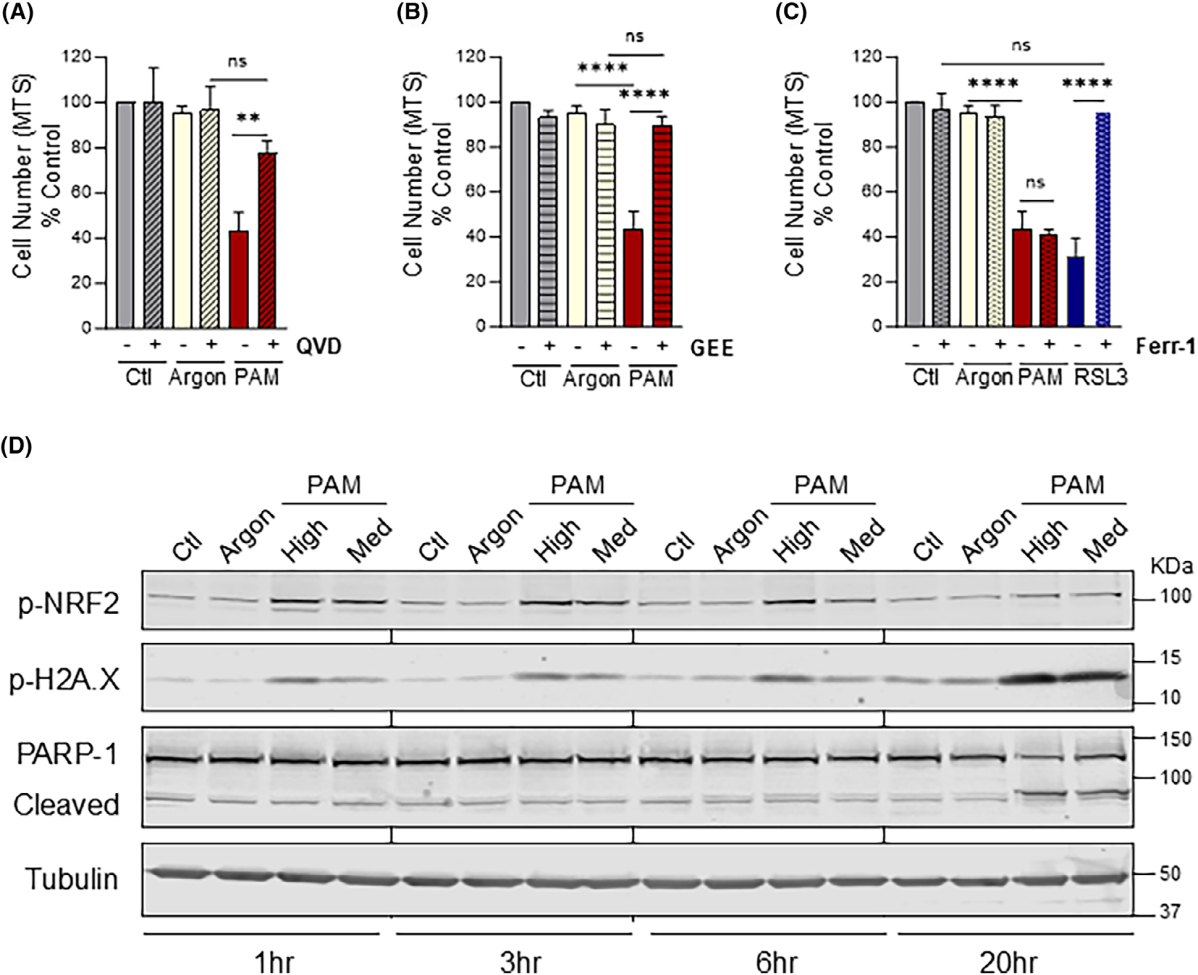
Plasma‐Activated Media (PAM) induces apoptosis in High‐Grade Serous Ovarian Cancer Cell (HGSOC) cell lines. (A) CAOV3 cells were pre‐treated with pan‐caspase inhibitor Q‐VD‐OPh (QVD) (50 μm) for 2 h prior to addition of control (Ctl), argon‐treated (Argon) or high‐dose PAM. The cell numbers were quantified by MTS after 24 h. (B) CAOV3 cells were pre‐treated with GEE for 2 h prior to addition of control, argon‐treated or high‐dose PAM. The cell numbers were quantified using 3‐(4,5‐dimethylthiazol‐2‐yl)‐5‐(3‐carboxymethoxyphenyl)‐2‐(4‐sulfophenyl)‐2H‐tetrazolium (MTS) viability assay after 24 h. (C) CAOV3 cells were pre‐treated with ferroptosis inhibitor Ferrostatin‐1 (Ferr‐1) (2 μm) for 1 h prior to addition of media control, argon‐treated media control, high‐dose PAM or pro‐ferroptotic agent RSL‐3 (2 μm). The cell numbers for A–C were quantified by MTS 24 h after treatment. MTS results are normalised to % media control and represented as mean ± Standard Deviation for three independent experiments (*n* = 3). Significance was determined by one‐way ANOVA with multiple comparisons (graphpad, prism v9). ** = *P* < 0.01, **** = *P* < 0.0001, ns, not significant. (D) CAOV3 cells were treated with media control, argon control, medium‐ or high‐dose PAM for the indicated times. Cleared lysate samples were subjected to SDS/PAGE and immunoblotting for: oxidative stress marker p‐NRF2 (Ser40); DNA damage marker p‐H2A.X (Ser139); caspase‐activation marker PARP‐1 (full length 116 kDa, cleaved 89 kDa); and loading control Tubulin. Blotting results are representative of three independent experiments (*n* = 3).

Reported cellular responses to PAM in ovarian cancer cell lines have ranged from induction of apoptosis, ferroptosis and autophagic cell death [19, 35, 36, 37, 38, 39]. As PAM‐treated cells appeared to have a rounded apoptotic morphology (Fig. 2E), an experiment was performed to rescue CAOV3 cells from apoptotic cell death by pre‐treating cells with a pan‐caspase inhibitor (QVD) prior to PAM treatment. Inhibition of caspase activity was able to rescue the viability in PAM‐treated CAOV3 cells to a level that matched the argon control (Fig. 3A). This strongly supports the hypothesis that PAM triggers a predominantly apoptotic response in CAOV3 cells.

PAM has previously been shown to overwhelm and inactivate the normal defences against oxidative assault at the plasma membrane, leading to catalase inactivation and depletion of the cellular global antioxidant, glutathione [40]. To test the contribution of oxidative stress in PAM‐induced cytotoxicity in the HGSOC setting, cells were co‐treated with a cell permeable glutathione precursor, glutathione ethyl ester (GEE) that is imported and rapidly converted to glutathione [41]. Treatment with high‐dose PAM for 24 h significantly decreased cell number of CAOV3 cells compared to the negative control treatments (Fig. 3B). Co‐treatment with GEE was able to completely rescue the PAM‐induced cytotoxicity to match the levels of the argon control (Fig. 3B), confirming that PAM promotes cell death through oxidative stress.

To test if the CAOV3 cytotoxicity was partially driven by PAM‐induced lipid peroxidation, CAOV3 cells were pre‐treated with the lipid antioxidant Ferrostatin‐1 (Ferr‐1) prior to application of PAM. While Ferr‐1 treatment was able to prevent the impacts of the ROS‐inducing, pro‐ferroptotic agent RSL‐3 [42], it failed to prevent PAM‐induced cell death (Fig. 3C). Together, these results suggest that PAM induces a predominantly pro‐apoptotic mode of cell death in CAOV3 cells that appears to be independent of lipid peroxidation or ferroptosis.

### 3.4 PAM induces oxidative stress and DNA damage

To dissect the mechanism of PAM‐induced HGSOC cell death, a panel of markers were analysed over a time course (1, 3, 6 and 20 h) in CAOV3 cells. A higher number and confluency of cells was required in this experiment to obtain sufficient sample for analysis. Cells grown in dishes were less sensitive to treatment and hence the more potent medium and high doses of PAM were chosen to assess biological response. The viability of the treated cells was confirmed by visual inspection (microscopy). Upon oxidative stress, the transcription factor Nuclear Factor erythroid‐derived 2‐like 2 (NRF2) is phosphorylated at Serine 40, allowing its activation and translocation to the nucleus to drive antioxidant gene expression [43]. PAM dose‐dependently increased the activation of Phospho‐NRF2 (p‐NRF2) compared to media and argon controls at 1, 3 and 6 h (Fig. 3D, top panel). The p‐NRF2 signal is terminated by the 20‐h timepoint indicating a swift and sustained oxidative response to PAM over several hours that is eventually downregulated. Similarly, an immediate dose‐dependent increase in the DNA damage double‐stranded break marker, phospho‐Ser139 Histone H2A variant (p‐H2A.X) [44], was observed with PAM treatment at 1 h that continued to increase at 3, 6 and the 20 h timepoints (Fig. 3D, second panel), suggesting a steady accumulation of double‐stranded breaks. Upon cell commitment to apoptosis, caspases are activated which causes cleavage of Poly(ADP‐Ribose) Polymerase (PARP‐1) to a smaller form (89 kDa) [45]. Cleaved PARP‐1 was detected in the PAM‐treated cells only at the 20‐h timepoint (Fig. 3D, third panel) suggesting that a pro‐apoptotic threshold was met. Taken together, the results suggest that PAM induces an immediate oxidative stress insult, with accumulation of DNA damage that culminates in inducing apoptosis by 20 h.

### 3.5 PAM induces mitochondrial membrane outer permeability

**Fig. 4:**
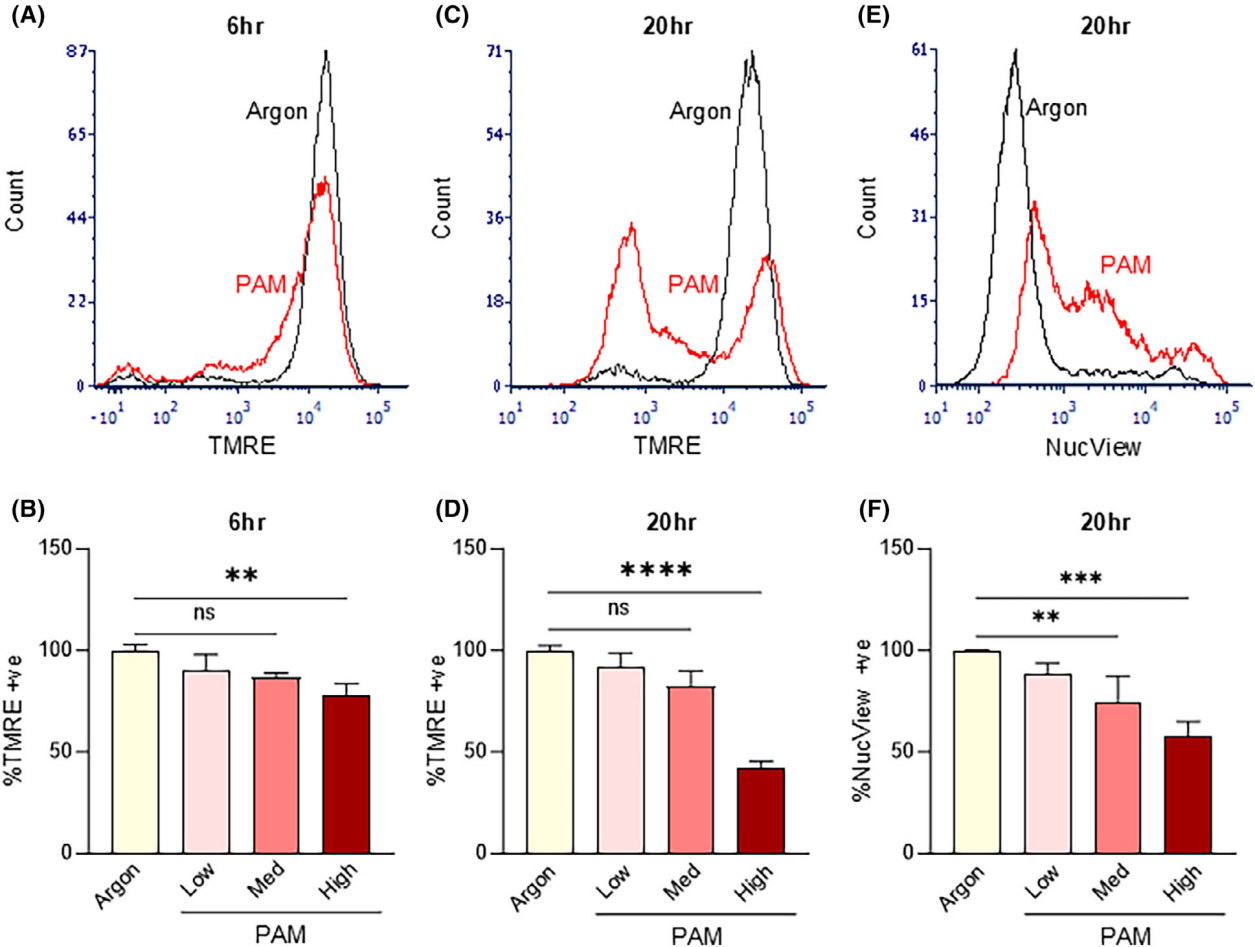
Plasma‐Activated Media (PAM) induces mitochondrial membrane outer permeability and apoptosis. CAOV3 cells were treated with argon‐treated media control, low‐, medium‐ or high‐dose PAM and were stained with either mitochondrial stain Tetramethylrhodamine ethyl ester perchlorate (TMRE) (A–D, *n* = 3) or activated caspase‐3 marker NucView (E, F, *n* = 2). (A) Representative histogram of number of positive cells (count) versus signal intensity (TMRE) at 6 h post‐treatment with argon control versus high‐dose PAM. (B) Quantified TMRE‐positive cell data at 6 h. (C) Representative histogram of number of positive cells (count) versus signal intensity (TMRE) at 20 h post‐treatment with argon control versus high‐dose PAM. (D) Quantified TMRE‐positive cell data at 20‐h. (E) Representative histogram of number of positive cells (count) versus signal intensity (NucView, caspase 3 activated) at 20 h post‐treatment with argon control versus high‐dose PAM. (F) Quantified NucView positive cell data at 20 h. (B–D, F) are represented as mean ± Standard Deviation. Significance was determined by one‐way ANOVA with multiple comparisons (graphpad, prism v9). ** = *P* < 0.01, *** = *P* < 0.001, **** = *P* < 0.0001, ns, not significant.

A key commitment step for oxidative stress‐induced apoptosis is disruption of mitochondrial membrane potential (ΔΨm) and mitochondrial outer membrane permeabilization (MOMP) that precedes the release of cytochrome C and activation of caspases [46]. The number of TMRE‐positive cells was quantified by flow cytometry as an indicator of mitochondrial membrane potential (Fig. 4, Fig. S1). CAOV3 cells were treated with argon control or low, medium and high‐dose PAM for 6 or 20 h. TMRE staining was significantly and dose‐dependently decreased by PAM at 6 h (Fig. 4A,B) and further decreased at 20 h (Fig. 4C,D). In follow‐up experiments using the NucView stain to detect activated caspase‐3, induction of apoptosis was only detected in CAOV3 cells treated with high‐dose PAM only at the later 20‐h timepoint (Fig. 4E,F), indicating a lag period between MOMP and commitment of the cells to apoptosis. Taken together, the western blotting (Fig. 3) and flow cytometry data (Fig. 4) suggest that PAM induces a sequential accumulation of oxidative stress and subsequent DNA damage followed by MOMP and then apoptosis in CAOV3 cells. The kinetics of the PAM‐induced apoptosis appeared to correlate with the accumulation of DNA damage as detected by p‐H2A.X (Fig. 3D).

### 3.6 PAM selectively induces apoptosis in primary tissue explant tumour cells

**Fig. 5:**
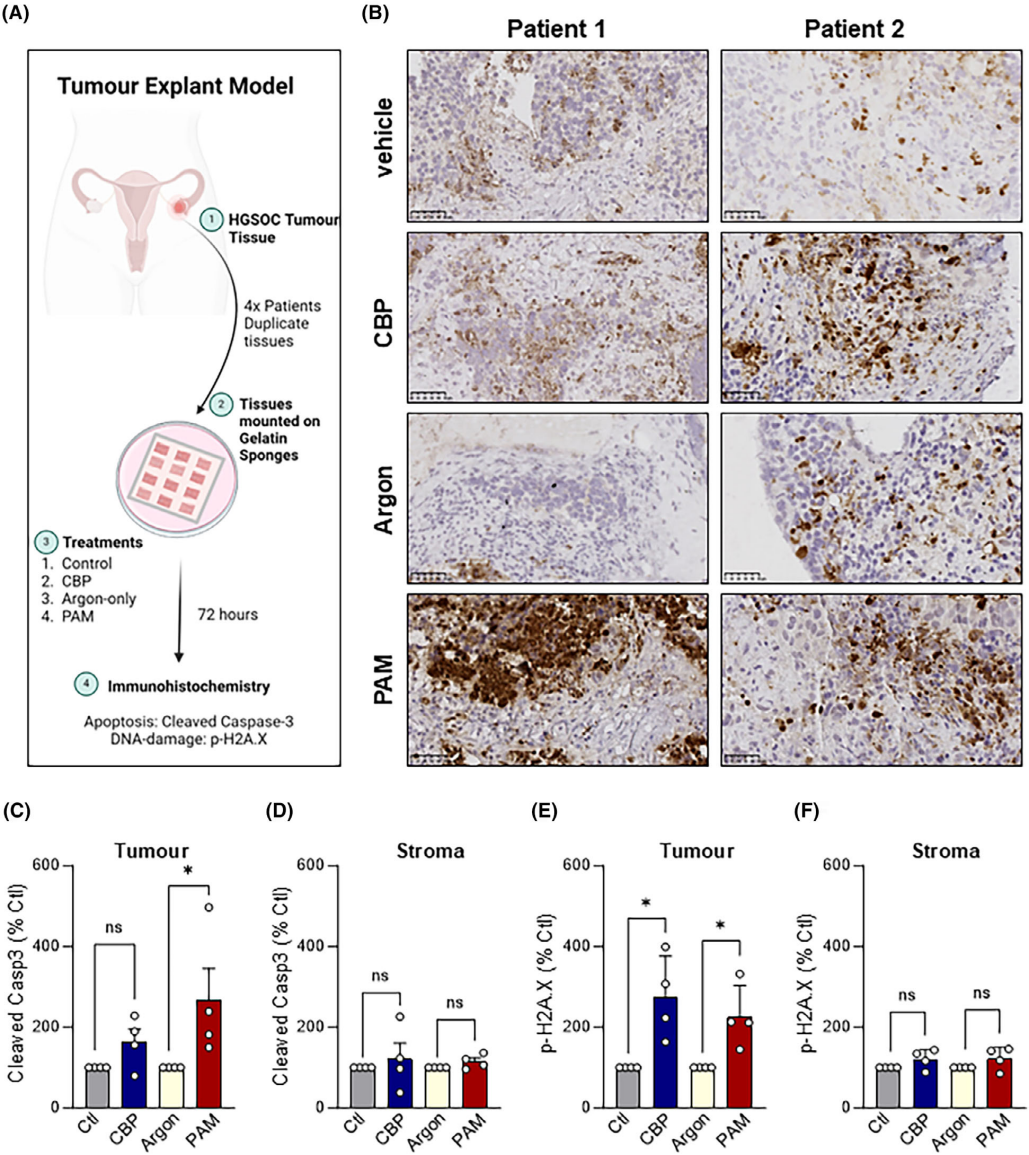
Plasma‐Activated Media (PAM) selectively promotes apoptosis in tumour cells in primary tissue explants. (A) Four High‐Grade Serous Ovarian Cancer Cell (HGSOC) patient tissue explants were mounted on gelatin sponges (*n* = 4, analysis in duplicate). The tissue explants were treated with control (media vehicle), CBP (100 μm in media), argon‐treated media or high‐dose PAM (duplicate tissue pieces) for 72 h. Samples were fixed and stained by Immunohistochemistry and counterstained with haematoxylin. (B) Representative images of Cleaved caspase‐3 staining in two patient samples to identify apoptotic cells (Scale bar indicates 50 μm). Cleaved caspase‐3‐positive cells were counted and represented as percentage control ± Standard Error of the Mean (SEM) in either (C) tumour cells or (D) stromal cells. p‐H2A.X positive cells in tumour were counted to identify cells with DNA damage and represented as percentage control ± Standard Error of the Mean (SEM) in either (E) tumour cells or (F) stromal cells (*n* = 4). Significance was determined by one‐way ANOVA with multiple comparisons (graphpad, prism v9). * = *P* < 0.05, ns, not significant.

An *ex vivo* tumour explant model derived from HGSOC patient tissue (Table S1) was used to test the feasibility of PAM in a more complex patient tumour setting [24]. The experiment involved treating cryopreserved primary tumour tissue segments on gelatin sponges with the test solutions for 72 h, and then fixing and analysing by immunohistochemistry (Summarised in Fig. 5A). The tumour tissue explants were treated with high‐dose PAM, argon‐treated media (negative control), CBP (positive control), and media alone (CBP vehicle control). Cleaved caspase‐3 and p‐H2A.X were used as markers of PAM‐induced apoptosis and DNA damage, respectively. High‐dose PAM induced a significant increase in cleaved caspase‐3 in tumour cells of the explant tissue that was comparable to the level achieved with the CBP positive control (Fig. 5B,C and Fig. S2). While the tumour cells were highly sensitive to PAM (Fig. 5C) we detected limited apoptotic (cleaved capase‐3) staining in the tissue stroma (Fig. 5D). Consistent with the results in cell‐based assays, tumour cell staining for the DNA damage marker p‐H2A.X was robustly increased with PAM treatment (Fig. 5E). The DNA damage response of PAM was comparable to the known DNA damaging agent and positive control CBP (Fig. 5E). Neither PAM nor CBP treatment had a significant impact on stromal cells in the explant tissue, with minimal p‐H2A.X staining detected in either treatment (Fig. 5F). Taken together, PAM treatment was able to induce a strong and selective pro‐apoptotic response in intact tumour tissue and induced a comparable response to chemotherapeutic agent CBP.

### 3.7 PAM reduces cell viability in primary ascites tumour cells from advanced stage chemoresistant patients

**Fig. 6:**
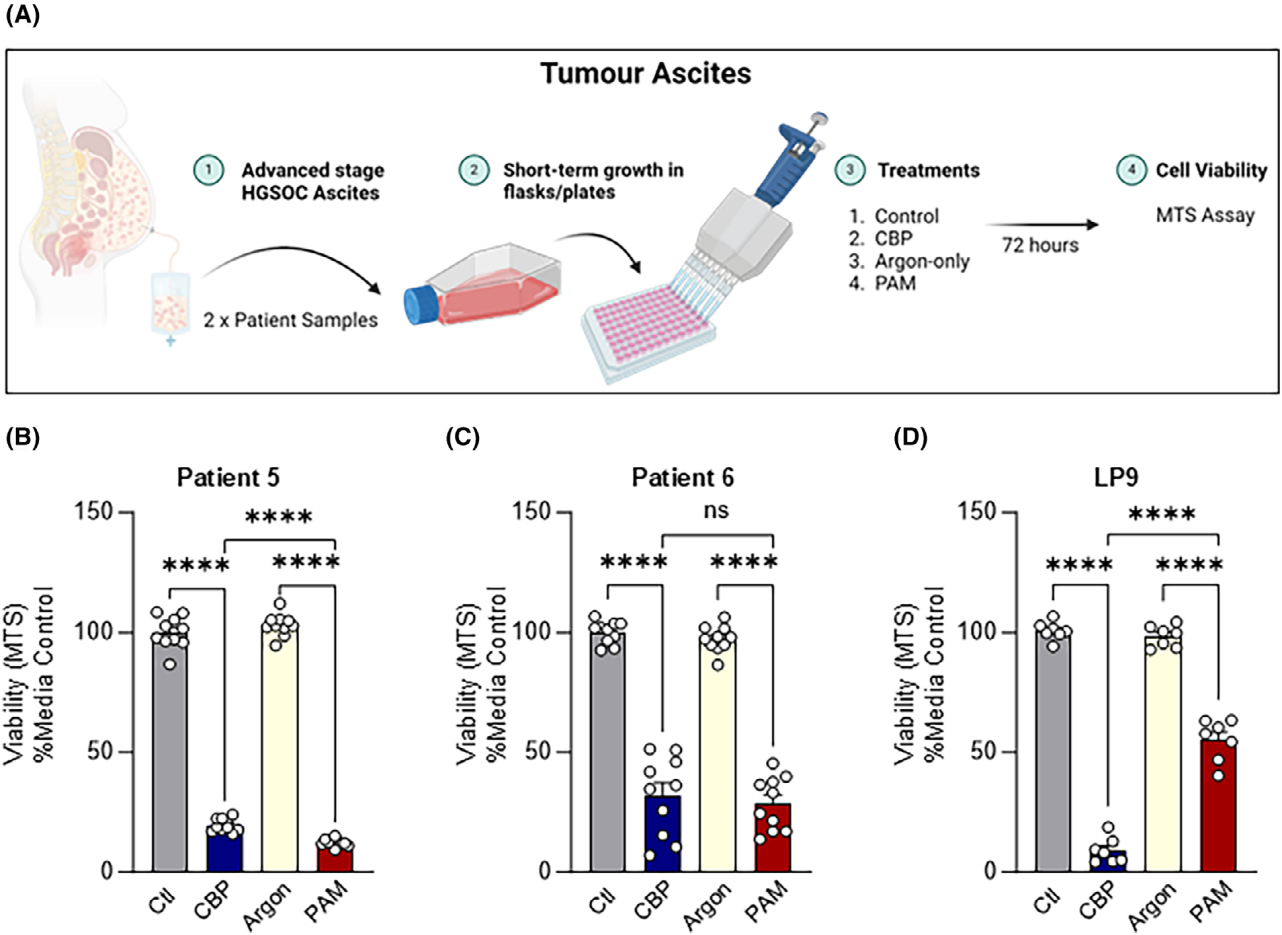
Plasma‐Activated Media (PAM) reduces cell viability in primary ascites tumour cells from advanced stage chemoresistant patients. (A) Ascites collected from two advanced stage chemoresistant High‐Grade Serous Ovarian Cancer Cell (HGSOC) patients were collected and plated in flasks. Outgrowth tumour cells were plated in 96‐well plates 24 h prior to treatment. The cells were treated with control (media vehicle, Ctl), chemotherapy carboplatin (CBP; 50 μm in media), Argon‐treated media (Argon) or high‐dose PAM (7 replicate wells). After 72 h viability of samples was estimated via 3‐(4,5‐dimethylthiazol‐2‐yl)‐5‐(3‐carboxymethoxyphenyl)‐2‐(4‐sulfophenyl)‐2H‐tetrazolium (MTS) viability assay. (B) Patient 5 (C) Patient 6 (D) Mesothelial cell line LP9 was included for comparison as a non‐cancer control. MTS results are normalised to % Media control and represented as mean ± Standard Deviation for seven‐replicates (*n* = 1). Significance was determined by one‐way ANOVA with multiple comparisons (graphpad, prism v9). **** = *P* < 0.0001, ns, not significant.

HGSOC patients with advanced stage disease develop a build‐up of metastatic tumour cells and fluid, known as ascites. The tumour cells in ascites grow in clusters called spheroids that attach to the peritoneal surfaces of the abdominal cavity and cause metastatic spread. To test whether PAM is effective at targeting this important disease niche, we collected and seeded low‐passage ascites tumour cells from two patients (Table S1). Peritoneal mesothelial cells (LP9) were included as a comparison to measure the relative sensitivity of the tumour cells versus the normal cells that line the abdominal cavity. In our focussed screen, the low‐passage tumour and mesothelial cells were treated with high‐dose PAM (compared to argon‐treated media as negative control) and CBP (positive control, compared to media‐alone) and the cell viability was estimated using MTS after 72 h (summarised in Fig. 6A). CBP treatment decreased cell viability to 20% in Patient 5, 32% in Patient 6 and 9% in LP9 cells, whilst PAM treatment decreased cell viability to 12% in Patient 5, 28% in Patient 6 and 55% in LP9 (Fig. 6B–D). PAM treatment had a significantly higher impact on Patient 6 primary cell viability compared to CBP (Fig. 6C). While both PAM and CBP treatment impacted LP9 viability, LP9 cells were more tolerable to PAM (with ~50% viability retention) compared to CBP (with less than 10% viability) treatments (Fig. 6D). To determine whether PAM treatment was antagonistic when used in combination with current standard of care agent, CBP, we tested PAM (medium dose) treatment alone or together with varying doses of CBP in CAOV3 and OV90 cells (Fig. S3). Combination treatment increased the cell death compared to either treatment alone, suggesting the treatment effects of PAM and CBP were additive.

## 4 Discussion

Treatment of HGSOC cells and patient tumours with PAM demonstrated a robust and selective anticancer effect. PAM efficacy in representative HGSOC cells [47] correlated with markers of oxidative stress and DNA damage that resulted in apoptotic cell death. Oxidative stress is known to trigger phosphorylation and activation of NRF2 to induce the expression of antioxidant and anti‐apoptotic proteins as a pro‐survival mechanism [48]. By 20 h, PAM‐treated cells displayed markers showing their commitment to apoptosis, with dephosphorylation and deactivation of NRF2, accumulation of DNA damage, loss of mitochondrial membrane potential and caspase activation. In line with the mechanistic findings of PAM treatment in HGSOC cell lines, tissue explants also displayed both DNA damage and caspase 3 activation. Finally, PAM treatment of two primary patient ascites tumour cell samples demonstrated sensitivity to PAM that was equivalent or better than CBP, the front‐line DNA damaging agent used for HGSOC treatment. PAM appeared to have minimal impact on surrounding stromal cells in the patient‐derived tumour explants and displayed a lower impact on peritoneal mesothelial cells than CBP and remained effective when used in combination with CBP. Overall, the data presented in this study provides evidence for PAM as a viable treatment of HGSOC worthy of further exploration.

The anticancer efficacy observed with PAM treatment in HGSOC cell lines aligns with previous work using other ovarian cancer cell lines with various subtypes and mutational profiles [19, 20, 37]. The present study has extended the efficacy of PAM to primary patient tissue explants and ascites cells. Of note, the representative HGSOC cells (CAOV3/OV90) and tissue samples harboured *TP53* driver mutations that are prevalent in ~ 97% of HGSOC patients [49]. While CAOV3 cells are reported to be highly representative of HGSOC [47], recent analysis inferred that OV90 cells may derive from mucinous ovarian cancer [50], the presence of a HGSOC‐specific gene fusion suggests that OV90 is more likely of HGSOC origin [51]. To address this limitation, we employed our tissue explant panel, containing samples from the immunoreactive/C2, differentiated/C4 and proliferative/C5 subtypes of HGSOC [30], and all displayed sensitivity to PAM treatment. While the panel lacked samples from the Mesenchymal/C1 subtype and had low numbers, this foundational work provides impetus to test PAM treatment in a larger tissue explant panel and elevates the clinical relevance of PAM treatment for epithelial ovarian cancers.

The PAM treatment appeared to selectively target cancer cells in patient tissues but had minimal impact on tumour stroma. PAM treatment also had minimal impact on normal mesothelial cells (LP9 cells). Thus, the PAM formulation in this study has similar selectivity for tumour cells as seen in previous studies using human ovarian surface epithelial cells [52, 53] and ovarian clear cell carcinoma mouse xenograft models [19, 20]. Taken together, the minimal impact on tumour stroma, mesothelial cells and ovarian surface epithelial cells, suggest that this formulation of PAM should be well tolerated in the peritoneal environment. Interestingly, unlike our RPMI‐based PAM, previous studies that have prepared PAM in Ringer's lactate solution have shown minimal induction of apoptosis but blockade of metastasis and increased survival in ovarian clear cell carcinoma mouse xenograft models [19, 20]. In contrast to other studies [20, 39], the PAM treatment in this study produced a high proportion of apoptotic cells which was inhibited with a pan‐caspase inhibitor but not with lipid antioxidants. Together, the results indicate that ferroptotic cell death was not a major pathway in our cell and tissue systems; albeit, this would need further validation in a larger panel of tissue explants and cell lines. The reason for the disparity may be due to the different ovarian cancer cell lines used or the preparation and dose of RONS within the PAM.

## 5 Conclusions

HGSOC is an aggressive disease with loss of normal TP53 control mechanisms such as cell cycle, DNA repair and apoptosis, which leads to genomic instability, copy number variations and tumour heterogeneity that drive disease progression and chemotherapeutic resistance [54, 55, 56]. Approaches that can exploit broad tumour cell vulnerabilities but minimise damage to normal tissues represent a new opportunity for standalone, combination or maintenance therapies in HGSOC. This study provides evidence that PAM treatment may be an effective strategy for selectively treating HGSOC and other types of EOC whilst minimising the impact on stromal cells and the peritoneal mesothelial lining. Key next steps for the confirmation of PAM therapy in HGSOC will be *in vivo* mouse models to investigate the impact of PAM not only on tumours but organs of the peritoneum and to investigate combination chemotherapy treatments in these models. It is envisaged that PAM treatment could be delivered to patients as a pre‐ or post‐surgery abdominal wash or incorporated into existing clinical paracentesis or HIPEC workflows [17] for therapeutic benefit.

## Conflict of interest

EJS declares a conflict of interest as co‐founder of Plasma 4. All other authors declare no conflict of interest.

## Author contributions

MRP, CR, NR and EJS were involved in conceptualization, formal analysis, investigation, methodology, analysis of data, data interpretation, visualisation, resources, funding acquisition, supervision and project administration. MRP, CR and EJS were involved in writing—original draft. LTD, RG, JT, CR, EJS, S‐HH, SKKC were involved in methodology, analysis of data, and data interpretation. MKO, NR, CR, EJS and MRP were involved in methodology, data interpretation, writing—editing, and review. All authors approved the final manuscript.

### Peer review

The peer review history for this article is available at https://www.webofscience.com/api/gateway/wos/peer‐review/10.1002/1878‐0261.13768.

## Supporting information


**Fig. S1.** PAM selectively promotes apoptosis in tumour cells in primary tissue explants.
**Fig. S2.** PAM selectively promotes apoptosis in tumour cells in primary tissue explants.
**Fig. S3.** PAM provides additive effects when used in combination with carboplatin.
**Table S1.** HGSOC patient explant clinical information.
**Table S2.** Normalised RNA‐sequencing data in tumour explants from patients 1 to 4.
**Table S3.** GSVA Molecular Subtype PrOTYPE classification in tumour explants from patients 1 to 4.
**Table S4.** Mutation Detection in tumour explants from patients 1 to 4.
**Table S5.** Mutation Detection in ascites samples from patients 5 to 6.

## Data Availability

The data that supports the findings of this study are available in Figs 1, 2, 3, 4, 5, 6 and the Supporting Information of this article. Raw data are available from the corresponding author melissa.pitman@adelaide.edu.au upon reasonable request.
