# Supplementary material for: Plasma‐activated media selectively induces apoptotic death via an orchestrated oxidative stress pathway in high‐grade serous ovarian cancer cells
Source: Mol Oncol. 2024 Dec 3;19(4):1170–87. doi: 10.1002/1878-0261.13768 (PMC11977661; doi:10.1002/1878-0261.13768)
Supplement: Supplementary file 1 — Fig. S1. PAM selectively promotes apoptosis in tumour cells in primary tissue explants. Fig. S2. PAM selectively promotes apoptosis in tumour cells in primary tissue explants. Fig. S3. PAM provides additive effects when used in combination with carboplatin. Table S1. HGSOC patient explant clinical information. Table S2. Normalised RNA‐sequencing data in tumour explants from patients 1 to 4. Table S3. GSVA Molecular Subtype PrOTYPE classification in tumour explants from patients 1 to 4. Table S4. Mutation Detection in tumour explants from patients 1 to 4. Table S5. Mutation Detection in ascites samples from patients 5 to 6. [file MOL2-19-1170-s001.zip › mol213768-sup-0001-Supinfo.pdf]

Supplementary Table 1: HGSOC Patient explant clinical information

Sample Type	Patient No	Stage, grade	Deleterious Mutations[S1]	Zygosity	Best-fit PrOTYPE Classification [S2]
**Primary HGSOC Ovarian Tumour Tissue**	1	Stage 4,<br>High grade	*TP53* Gly244Asp<br>*TP53* Pro72Arg<br>*BRCA1* Ser471Gly*	*Heterozygous*<br>*Homozygous*<br>*Homozygous*	Proliferative
	2	Stage 3,<br>High grade	*TP53* Tyr126Cys<br>*TP53* Pro72Arg<br>*BRCA1* Ser471Gly*<br>*BRCA1* Lys118Arg*	*Homozygous*<br>*Homozygous*<br>*Homozygous*<br>*Homozygous*	Proliferative
	3	Stage 2,<br>High grade	*TP53* Cys275Tyr<br>*TP53* Pro72Arg	*Homozygous*<br>*Homozygous*	Differentiated
	4	Stage 3,<br>High grade	*TP53* Pro72Arg<br>*BRCA1* Ser471Gly*<br>*BRCA1* Lys118Arg*	*Heterozygous*<br>*Homozygous*<br>*Homozygous*	Immunoreactive
**Primary Chemo-resistant Ascites Cells from HGSOC patients**	5	Stage 3,<br>High grade	*KRAS* Gly12Asp	*Heterozygous*	NA
	6	Stage 4,<br>High grade	*TP53* R213*stop*	*Heterozygous*	NA

*indicate variants of unknown significance [S3]; NA = analysis not applicable to ascites samples. Raw data is included in the SupportingData.xlsx file.

Supplementary References:

[S1] Talhouk A, George J, Wang C, Budden T, Tan TZ, et al. (2020) Development and Validation of the Gene Expression Predictor of High-grade Serous Ovarian Carcinoma Molecular SubTYPE (PrOTYPE). Clin Cancer Res 26: 5411-5423 doi:10.1158/1078-0432.CCR-20-0103

[S2] McLaren W, Gil L, Hunt SE, Riat HS, Ritchie GR, et al. (2016) The Ensembl Variant Effect Predictor. Genome Biol 17: 122 doi:10.1186/s13059-016-0974-4

[S3] Karami F and Mehdipour P (2013) A comprehensive focus on global spectrum of BRCA1 and BRCA2 mutations in breast cancer.

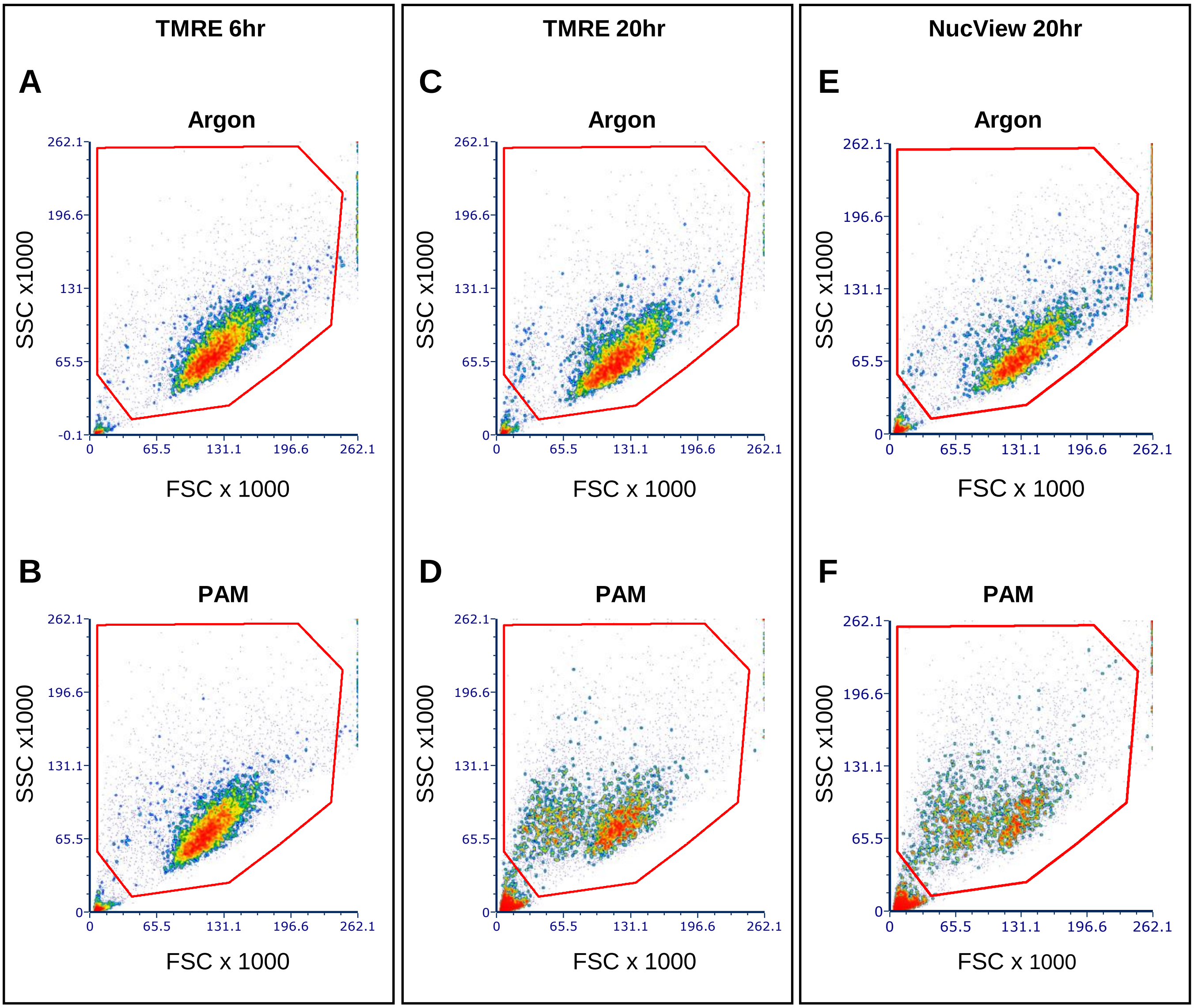

Supplementary Figure 1: Plasma Activated Media (PAM) selectively promotes apoptosis in tumour cells in primary tissue explants

CAOV3 cells were treated with argon-treated media control, low, medium or high dose PAM and were stained with either mitochondrial stain Tetramethylrhodamine ethyl ester perchlorate (TMRE) (A-D, n=3) or activated caspase-3 marker NucView (E-F, n=2). Representative scatterplots of Forward Scatter (FSC) and Side Scatter (SSC) showing gating of viable cell populations of TMRE-stained cells post treatment of Argon or PAM-treated at 6 hours (A-B) or 20-hours (C-D) or NucView-stained post treatment of Argon or PAM-treated at 20 hours (E-F).

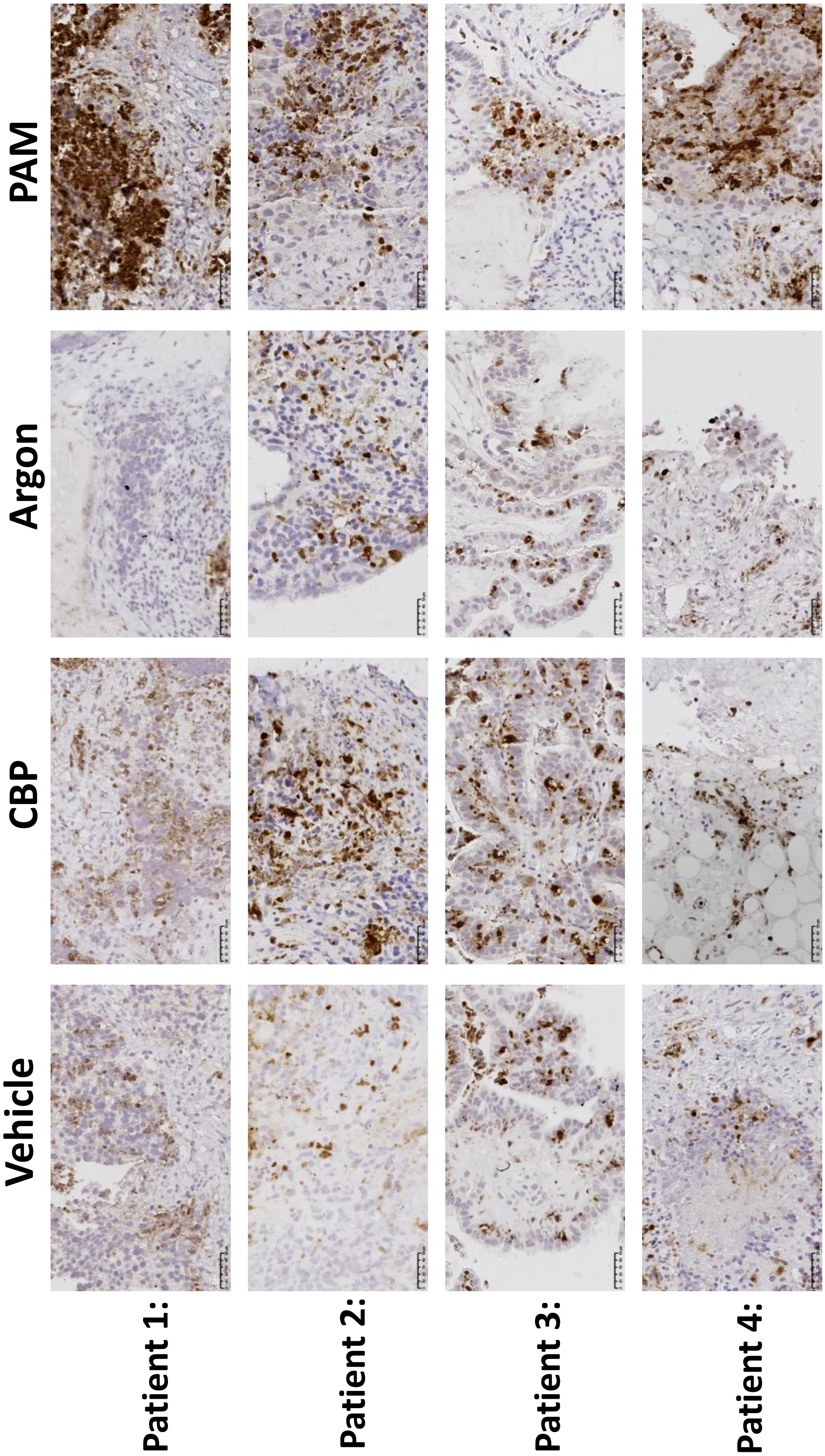

Supplementary Figure 2: Plasma Activated Media (PAM) selectively promotes apoptosis in tumour cells in primary tissue explants

Four High-Grade Serous Ovarian Cancer Cell (HGSOC) patient tissue explants were mounted on gelatin sponges (in duplicate). The tissue explants were treated with control (media vehicle), carboplatin (CBP) (100 μM in media), argon-treated media (Argon) or high dose PAM (duplicate tissue pieces) for 72 hours. Samples were fixed and stained by Immunohistochemistry and counterstained with hematoxylin. Representative images of Cleaved-caspase 3 staining in patient samples to identify apoptotic cells (Scale bar indicates 50 μm).

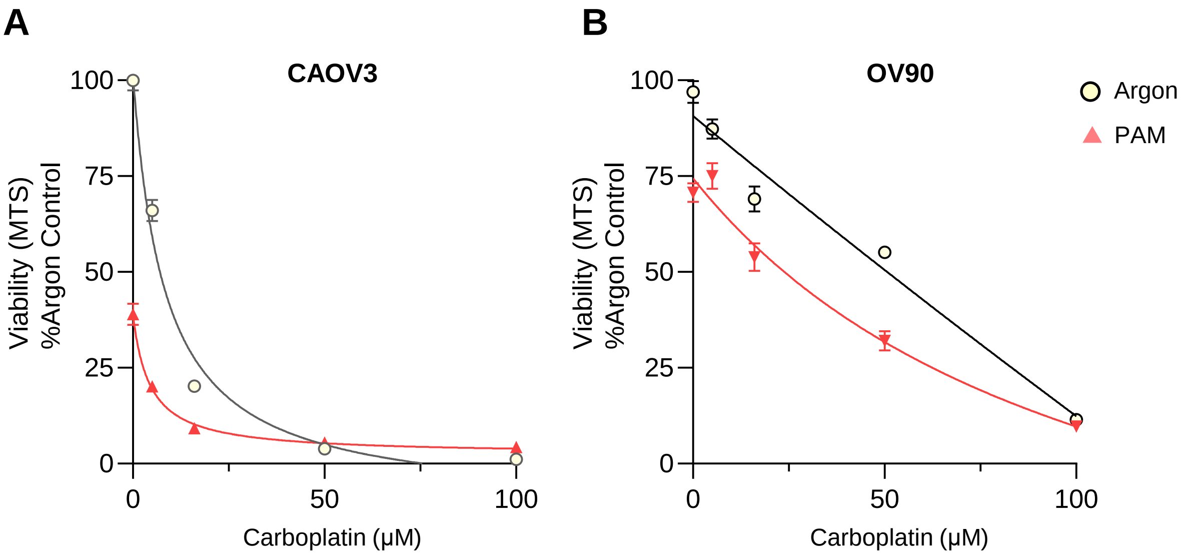

Supplementary Figure 3: Plasma Activated Media (PAM) provides additive effects when used in combination with carboplatin

A) CAOV3 or B) OV90 cells were treated with argon control (Argon) or medium dose PAM plus varying doses of carboplatin (0, 5, 15, 50, 100 μM). After 72 hours of treatment cell number was quantified by 3-(4,5-dimethylthiazol-2-yl)-5-(3-carboxymethoxyphenyl)-2-(4-sulfophenyl)-2H-tetrazolium (MTS) viability assay. Results are normalised to % Argon control and represented as mean ± Standard Error of the Mean (SEM) for five-replicates, n=1. Dose-curves were estimated using Non-linear Regression ([Inhibitor] vs Response (three parameters) (Graphpad Prism).
